# General prognostic models may neglect vulnerable subgroups in ANCA-associated vasculitis

**DOI:** 10.1007/s40620-023-01726-5

**Published:** 2023-09-28

**Authors:** Martin Russwurm, Birgit Kortus-Goetze, Tanja Maier-Giebing, Hermann-Josef Groene, Joachim Hoyer

**Affiliations:** 1 Centre for Internal Medicine, Renal Division, University Hospital Marburg, Marburg, Germany; 2 Institute of Pharmacology, Philipps-University, Marburg, Germany; 3 Medical Faculty, University of Heidelberg, Heidelberg, Germany

**Keywords:** ANCA-associated vasculitis, Renal replacement therapy, Elderly, Mortality, Renal recovery, Survival, Outcome

## Abstract

**Background:**

ANCA-associated vasculitis is an organ and life-threatening disease with the highest incidence in elderly patients. However, few studies have focussed on characteristics and treatment outcomes in a direct comparison of elderly and younger patients.

**Methods:**

In a retrospective, single-centre, renal biopsy-cohort, patients were dichotomized by age ≥ 65 years to analyse baseline clinical, histological, laboratory and immunological characteristics and outcome differences in elderly and younger patients as regard to mortality, renal recovery from dialysis and eGFR after two years.

**Results:**

In the biopsy registry, *n* = 774 patients were identified, of whom 268 were ≥ 65 years old. Among them, ANCA-associated vasculitis was the most prevalent kidney disease (*n* = 54 ≈ 20%). After a follow-up of 2 years, overall mortality was 13.4%, with 19% and 4% in patients ≥ and < 65 years of age, respectively. While 41% of elderly and 25% of younger patients were dialysis-dependent at the time of biopsy, renal recovery was achieved in 41% and 57% of patients, respectively. The accuracy of prediction differed significantly between the whole cohort and elderly patients as regard to mortality (sensitivity 46% vs. 90%, respectively) and between younger and elderly patients as regard to eGFR (*r*^2^ = 0.7 vs. 0.46, respectively). Age-group-wise analysis revealed patients above 80 years of age to have particularly dismal renal outcome and survival.

**Conclusion:**

In our cohort, ANCA-associated vasculitis is the single most frequent histopathological diagnosis among the elderly patients in our cohort. Elderly and younger patients have comparable chances of recovering from dialysis-dependent renal failure, with comparable residual independent kidney function after two years. This study suggests (1) relevant predictors differ between age groups and hence (2) models involving all patients with ANCA-associated vasculitis neglect important features of vulnerable subgroups, i.e., patients above 80 years old.

**Graphical abstract:**

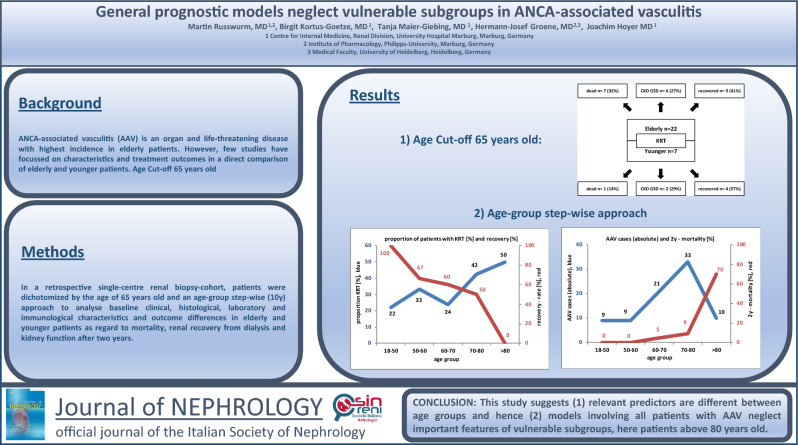

**Supplementary Information:**

The online version contains supplementary material available at 10.1007/s40620-023-01726-5.

## Introduction

Anti- neutrophil cytoplasmic antibody (ANCA)-associated vasculitides are autoimmune diseases that occur in all age groups with a reported peak incidence between 65 and 74 years of age [1, 2]. Although there are recommendations regarding the treatment of elderly patients with ANCA-associated vasculitis (AAV), studies addressing this issue are few [1, 3–7]. These studies show marked heterogeneity in diagnostic standard, case definition, treatment and outcome. Particular emphasis is given here to the issue of treatment indication in the elderly.

Earlier data highlighted infectious complications of standard immunosuppression and the possibility of a “futility threshold” of therapeutic attempts in this cohort [8–10]. ANCA-associated vasculitides with renal involvement are by definition classified as severe disease [11]. This study aims to add evidence to a growing body of data to deploy adjusted guideline-directed therapy to elderly patients with severe AAV by direct comparison of characteristics and outcomes of patients below and above 65 years of age in a retrospective observational study design.

### Subjects and methods

#### Patients’ data, lab samples and renal biopsies

We retrospectively analysed histopathological data of all patients aged ≥ 18 years that underwent a renal biopsy from 2001 to mid 2017 at the university hospital Marburg, Germany.

All biopsies were processed by light microscopy, immunohistochemistry and electron microscopy.

In-depth data acquisition was performed for the sub-cohort of pauci-immune necrotizing glomerulonephritis, indicative for ANCA-associated renal vasculitis. In particular, diagnosis was based upon renal pathology findings, ANCA and target antigen positivity and the clinical picture. The degree of AAV was defined by either positivity for perinuclear ANCA plus myeloperoxidase-antibody (p-ANCA/MPO) for MPO-AAV and cytoplasmic ANCA plus proteinase-3-antibody (c-ANCA/PR3) for PR3-AAV, respectively. Other necrotizing glomerulopathies, i.e., anti-glomerular basement membrane glomerulohephritis (even if ANCA positive) and necrotizing IgAN were excluded from analyses, as were patients with eosinophilic granulomatosis with polyangiitis. Patients’ data were analysed for two years, as of the date of initial biopsy. In the AAV cohort the number of normal, scarred and necrotic glomeruli were assessed, as well as the interstitial fibrosis and tubular atrophy (IF/TA) quantified as a proportion of the entire tubulointerstitium. Normal glomeruli are defined as those without AAV-associated changes. Necrotic glomeruli are defined as those with any fresh necrosis detectable, and scarred glomeruli are defined as those showing any form (segmental or global) of scarification. As regard to vascular changes, we semiquantitively assessed nephrosclerosis as well as arteriitis. The ANCA renal risk score was estimated precisely as published elsewhere [12]: In brief, eGFR, number of normal glomeruli and IF/TA are weighed to assign patients to low, medium or high risk probability of developing end-stage kidney disease after 3 years.

Medical records were reviewed for the presence of pre-existing comorbidities. Chronic disease (COPD, heart failure, chronic kidney disease [CKD], diabetes mellitus, arterial hypertension, coronary heart disease) as well as history of stroke and/or cancer were rated one point each. All blood and urine samples evaluated for statistical analysis were taken simultaneously at the time of the renal biopsy, and subsequently, one and two years after index admission for follow-up analysis, either from hospital files or as reported by the primary care physician or nephrologist. Initial eGFR was estimated by the abbreviated MDRD-formula and CKD-EPI formula. For further analyses the abbreviated MDRD-formula was used.

Treatment of AAV was performed according to guideline recommendations [11]. In general, plasma exchange was performed in the presence of severe organ damage, i.e., the need for kidney replacement therapy (KRT) and/or when serum creatinine increased above 5.7 mg/dl or the patients suffered from diffuse alveolar haemorrhage. In modulation of the MEPEX trial, 5–7 plasma exchanges were performed, and were generally substituted with fresh frozen plasma in awareness of coagulation disturbances associated with albumin-only substitution in patients at increased bleeding risk. Importantly, plasma exchange in our patients was accompanied by high-dose steroid therapy.

Immunosuppressive induction therapy comprised high dose i.v. (250–500 mg daily) methylprednisolone (d1-3), followed by a tapering regimen for prednisolone: 100 mg p.o. (d4-d7), reduced to 0.75 mg/kg body weight at week 2 with a slow taper to a maintenance dose of 0.04 mg/kg body weight at one year and for the following second year. The majority of patients received weight-, age-, and eGFR-adjusted i.v. cyclophosphamide every three weeks for a total of 6 administrations until remission in slight modulation (in terms of infusion intervals) of the protocol of the CYCLOPS-trial [13]. Alternatively, Rituximab could be administered at a dosage of 375 mg/m^2^ body surface area weekly for four weeks [14].

Consecutively, standard maintenance immunosuppression comprised azathioprine (AZA) or mycophenolate (MMF) plus a prednisolone-tapering regimen as described above. All patients received co-medication with MESNA after every CYC-therapy, pantoprazole and vitamin D supplementation (1000 IU once daily) added to steroid therapy, as well as cotrimoxazole as prophylaxis for *P. jirovecii* infection. Alternatively, Rituximab (500 mg absolute) could be administered for maintenance therapy at months 6, 12 and 18 in accordance with the protocol of the MAINRITSAN trial [15].

### Statistical methods

At a significance level of 5%, statistical analyses were carried out as specified in the respective data set. For parametric data, Student’s *t*-test was performed. In case of violation of the homogeneity of variance prerequisite or when making comparison of medians, a Wilcoxon rank sum test was applied. Comparison of dichotomous data was performed using Fisher’s exact test. For survival analysis a Kaplan–Meier estimate was calculated and significance calculated by Cox log-rank test.

Missing data were omitted for analyses. This holds especially true for ratios and statistical analyses in case of drop-out by censoring (or loss of follow-up) and patient death and/or end-stage renal disease i.e., for calculation of mean eGFR in the follow-up.

Univariate and multivariate linear regression models were used for analysis of different initial parameters for associations with residual renal function. The parameters analysed were: age, female sex, haemoglobin level, albumin level, eGFR, need for KRT, AAV type (MPO-AAV/PR3-AAV), C-reactive protein, comorbidity score (as referenced above), number of necrotic/scarred/unaffected glomeruli, number of necrotic/scarred glomeruli as proportion of unaffected glomeruli and interstitial fibrosis/tubular atrophy. We report the best-fit models, suggesting that also non-significant variables are implemented in the model if adding predictive value. Associations of patient characteristics with the dichotomous outcomes “chronic kidney disease treated by dialysis” (CKD G5D, according to the latest KDIGO nomenclature) and “death” were analysed by binary logistic regression models. The variables implemented in the definitive model are stated in the respective text passage or figure legend.

## Results

### Biopsy cohort

**Fig. 1:**
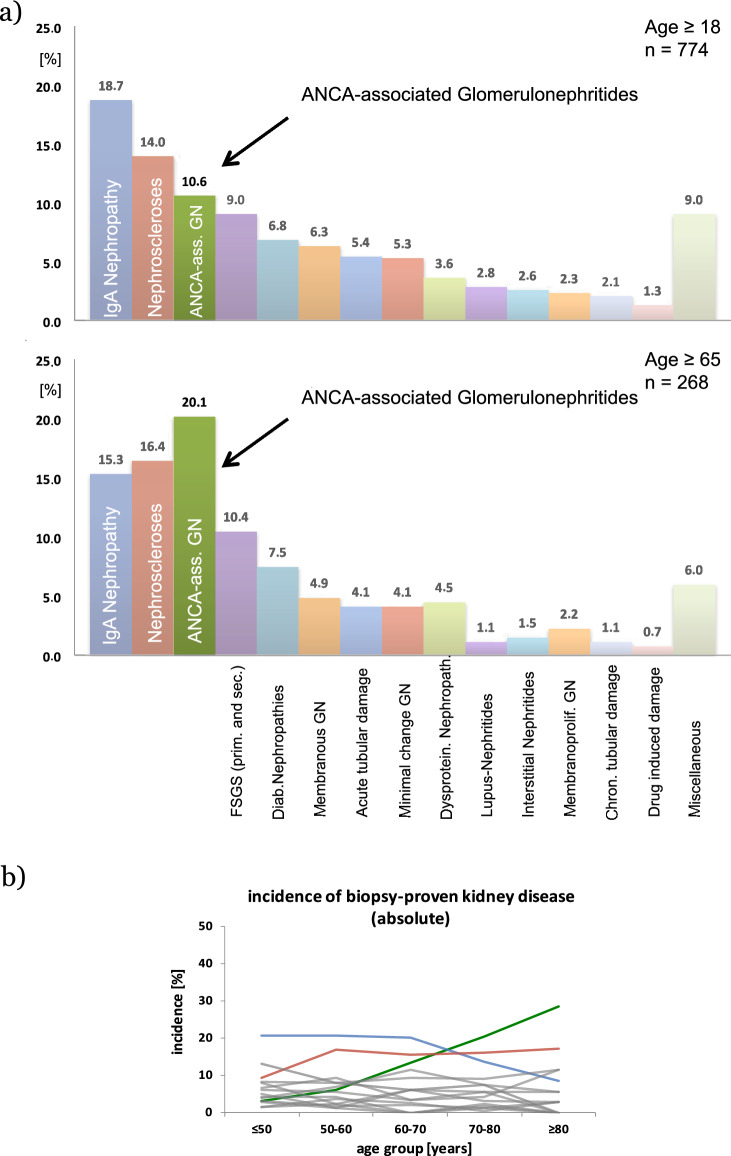
**a** Distribution of incidental biopsy-proven renal diseases. The upper figure depicts the distribution of renal pathology findings in the entire cohort. The lower figure shows the respective distribution in patients aged 65 years or older. Miscellaneous comprises, among others, glomerulonephritis with detection of antibodies against the glomerular basement membrane, endocapillary (proliferative) glomerulonephritis, fibrillary glomerulonephritis, mesangiocapillary glomerulonephritis, Alport‘s disease nephropathy, thrombotic micoangiopathies (including atypical hemolytic uremic syndrome, malignant hypertension), kidney infarction, cholesterol embolism, renal crisis in sclerodermia, Fabry‘s disease, sarcoidosis, renal lymphoma, nephronophthisis, nephrocalcinosis, Bunya-Viridae nephritis and others. **b** Incidence as a fraction of whole cohort of biopsy-proven kidney disease in respective age groups. (blue line depicts IgAN, red line indicates nephrosclerosis, green line depicts ANCA-ass. vasculitis)

A total of 774 biopsies were analysed, with IgA nephropathy being the most common biopsy-proven renal disease (18.7%), followed by nephrosclerosis (i.e. hypertensive nephropathy, 14%) and pauci-immune necrotizing glomerulonephritis, accounting for 10.6% of all biopsies (Fig. 1a upper part). A total of 268 patients were ≥ 65 years of age. In this sub-group, pauci-immune glomerulonephritis accounted for 20.1% of diagnoses. Thus, AAV was the most common biopsy-proven kidney disease in elderly patients (Fig. 1a lower part). The relative incidence of AAV in this renal biopsy cohort rose with age (Fig. 1b). In the whole cohort, 82 patients had biopsy-proven AAV: 54 patients were ≥ 65 years of age and 28 patients were < 65 years of age.

### Baseline characteristics of the AAV-cohort

For comparison of prerequisites and outcomes, all patients with a diagnosis of renal AAV were dichotomized by age 65 years into two groups with 54 patients in the elderly and 28 patients in the younger cohort. Baseline characteristics on the day of diagnosis are shown in Table 1. The median age in the elderly was 75 years, thus 20 years higher than the median age among the younger patients. Elderly patients had a higher comorbidity score than younger patients (1.7 vs. 0.8; *p* < 0.001). There was no statistical difference in gender, ANCA specificity, albumin, haemoglobin, and C-reactive protein concentrations (Table 1). Histologically, there was no difference in glomerular necrotic lesions, but elderly patients tended to have more frequent (30% (16/54) vs. 11% (3/28)) and pronounced nephrosclerosis and IF/TA at baseline (Table 1 For individual assignment of pathology findings refer to Figure S1).

**Table 1:** Baseline characteristics of AAV cohort

			Whole cohort	Elderly (≥ 65 years)	Younger (< 65 years)	*p*
General/clinical						
Number n			82	54	28	
Observation period	[Years]	Median (IQR)	2.01 (1.95–2.07)	2.00 (1.89–2.07)	2.01 (1.96–2.08)	0.864
Age at diagnosis	[Years]	Mean ± SD	67.1 ± 14.0	75.1 ± 5.8	51.6 ± 12.2	< 0.001
		Median (IQR)	71 (60–77)	75 (71–79)	55.5 (46–60)	< 0.001
Female	[Dimensionless]	[*n*; %]	39 (48%)	26 (48%)	13 (46%)	> 0.99
Comorbidity index	[Dimensionless]	Mean ± SD	1.4 ± 1.1	1.7 ± 1.0	0.8 ± 1.0	< 0.001
Immunological						
PR3-AAV	[Dimensionless]	[*n*; %]	30 (37%)	16 (30%)	14 (50%)	0.092
MPO-AAV	[Dimensionless]	[*n*; %]	47 (57%)	34 (63%)	13 (46%)	0.166
ANCA-negative	[Dimensionless]	[*n*; %]	5 (6%)	4 (7%)	1 (4%)	0.656
General laboratory						
Serum creatinine conc.	[mg/dl]	Mean ± SD	4.62 ± 2.93	4.83 ± 2.42	4.33 ± 3.73	0.475
eGFR (MDRD)	[ml/min]	Mean ± SD	21.7 ± 23.4	18.5 ± 19.3	28.0 ± 29.5	0.087
		Median (IQR)	11.9 (8.1–27.9)	10.3 (7.6–23.2)	21.4 (8.6–36.2)	0.054
eGFR (CKD-EPI)	[ml/min]	Mean ± SD	21.6 ± 23.7	18.0 ± 20.0	28.9 ± 29.0	0.051
		Median (IQR)	11.3 (7.4–26.8)	9.8 (7.0–17.6)	21.8 (8.5–38.9)	0.029
C-reactive protein	[mg/l]	Mean ± SD	130 ± 100	126 ± 102	141 ± 98	0.528
Albumin	[g/l]	Mean ± SD	27 ± 7	27 ± 7	28 ± 7	0.642
Haemoglobin	[g/dl]	Mean ± SD	9.9 ± 1.7	9.9 ± 1.8	9.8 ± 1.6	0.874
MCV	[fl]		85 ± 5	86 ± 5	85 ± 5	0.433
MCH	[pg]		28 ± 2	29 ± 2	28 ± 2	0.638
Histopathologic						
Glomeruli	Number	Median ± SD	12 (8–17)	11 (7–14)	14 (10–18)	0.090
Glomeruli (necrotic)	[%]	Mean ± SD	40 ± 26	38 ± 26	45 ± 25	0.231
Glomeruli (scared)	[%]	Mean ± SD	27 ± 23	29 ± 24	24 ± 22	0.432
Glomeruli (normal)	[%]	Mean ± SD	32 ± 23	34 ± 23	31 ± 25	0.587
IF/TA §	[%]	Mean ± SD	23 ± 25	27 ± 14	14 ± 20	0.026
Nephrosclerosis		[*n*; %]	19 (23%)	16 (30%)	3 (11%)	0.060
Nephrosclerosis	[sq]	Mean ± SEM	0.63 ± 0.13	0.83 ± 0.18	0.25 ± 0.14	0.029

Test for statistical significance between elderly and younger patients

§ IF/TA interstitial fibrosis and atrophy–histological measure for chronic injury

Serum creatinine at presentation did not differ between groups. Estimated GFR was lower in the elderly (MDRD-eGFR: 10.3 vs. 21.4 ml/min, *p* = 0.051; CKD-EPI-eGFR: 9.8 vs. 21.8 ml/min, *p* = 0.029).

### Therapy

There was no difference in the percentage of patients receiving standard vs. alternative induction and maintenance therapy in the respective age groups (supplementary Tab. S1). There was one patient in the elderly group who did not receive induction therapy. In the maintenance phase, there was a tendency to treat elderly patients preferably with azathioprine instead of mycophenolate, which was more prevalent in younger patients.

Overall, 40% of patients (*n* = 33) were treated with plasma exchange in the whole cohort, with no statistical difference in plasma exchange utilization between elderly (46% *n* = 25/54) and younger patients (29% *n* = 8/28; *p* = 0.121). In patients receiving kidney replacement therapy (KRT), 72% (*n* = 21/29) also received plasma exchange. In these patients plasma exchange utilization did not differ between elderly (68% *n* = 15/22) and younger patients (86% *n* = 6/7; *p* = 0.817) receiving KRT. In patients not receiving KRT, plasma exchange was applied in 23% (*n* = 12/53), with a tendency to treat elderly patients more frequently with plasma exchange (31% *n* = 10/32) than younger patients (10% *n* = 2/21; *p* = 0.064).

### Survival

**Fig. 2:**
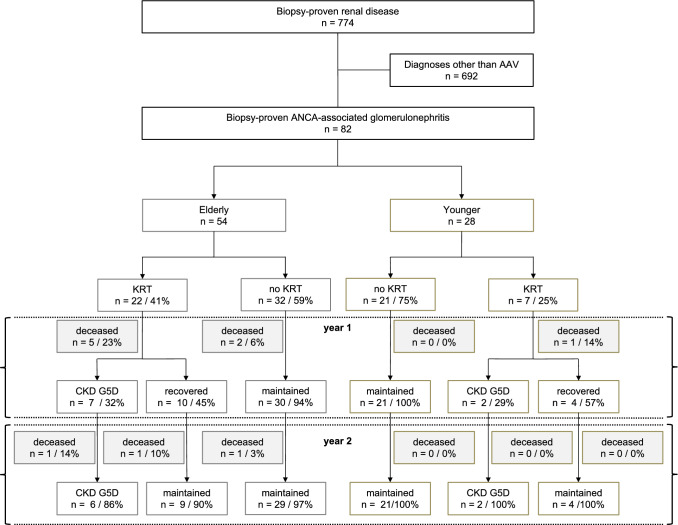
Flow-diagram of screening cohort and AAV patients’ clinical courses; highlighting end stage kidney disease and death. Percentage rates relate to the absolute number of cases in the respective level above. KRT kidney replacement therapy; CKD G5D end stage renal disease

In the follow-up period of two years a total of eleven patients died (13.4% *n* = 11/82), ten in the elderly (18.5% *n* = 10/54), and one in the younger group (3.6% *n* = 1/28, *p* = 0.059, flow-diagram of outcomes depicted in Fig. 2).

**Fig. 3:**
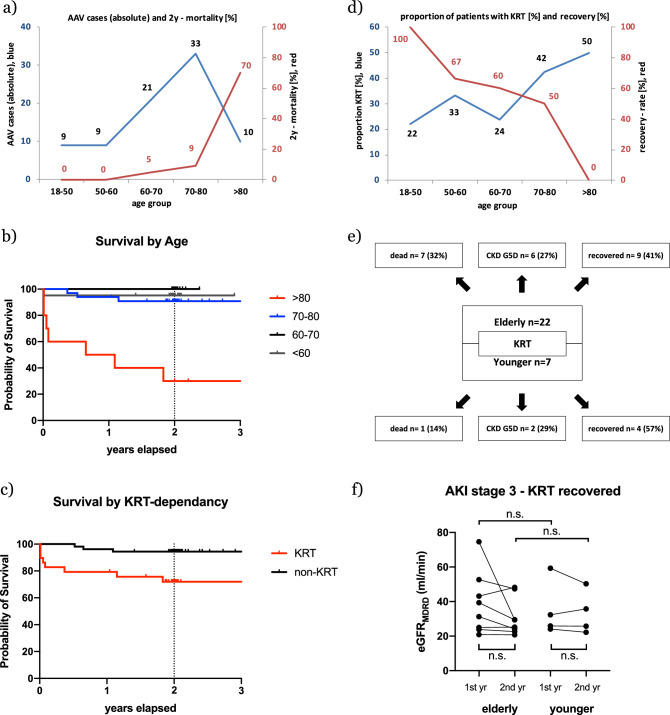
**a** Absolute cases of AAV (blue line) and two-year mortality (red line) in the respective age group. Kaplan–Meier plot of survival for the respective age groups (**b**) and the KRT and non-KRT group (**c**), respectively. **d** Proportion of patients with initial KRT dependency (blue line) and respective recovery rate (red line). **e** Descriptive outcome of patients with initial KRT-dependency. **f** Residual renal function in patients that recovered from initial KRT-dependency in the respective age groups

The median age of deceased patients was 79 years, with a median comorbidity score of 2.3 as compared to 1.2 in survivors (*p* = 0.004). The median time to death was 189 days. Five patients (45%) died within one month after diagnosis, including the one patient in the younger group. Patients that survived this initial phase, survived for a median time of 336 days. Displaying absolute case numbers and mortality rates in the respective group in ten-year steps identifies patients aged ≥ 80 years as a group with particularly dismal survival and a mortality rate of 70% (*n* = 7/10; Fig. 3a and b) in two years. The difference in mortality between those patients (70% *n* = 7/10) and patients < 80 years of age (6% *n* = 4/72) was significant (p < 0.001). Among the deceased patients, 73% (*n* = 8/11) received KRT. Mortality rate of patients that received KRT was significantly higher than the mortality rate of patients that did not receive KRT (27.5% *n* = 8/29 vs. 5.6% *n* = 3/53, respectively; *p* = 0.005; Fig. 3c).

**Fig. 4:**
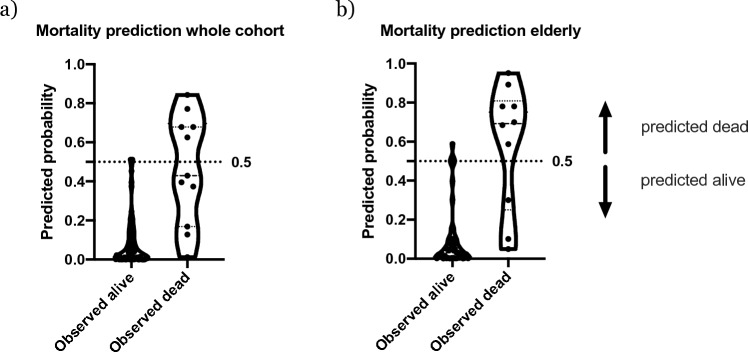
Truncated Violin plot of predicted and observed events (death within 2 years follow-up) in the whole cohort (**a**) and elderly patients (**b**); cut off (differentiation) probability 0.5. Independent variables: age, initial KRT-dependency. The one additional (younger) deceased patient alters the model, so that probability for every data point is not equal in both models

In a univariate regression analysis, KRT-dependency, age and comorbidity score were significantly associated with mortality (Tab. S3). Due to the occurrence of only one event in the younger group, statistics were not calculated. In a multivariate regression model, only KRT-dependency and age were independently associated with two-year-mortality in both the elderly and the whole cohort. The goodness of fit however, showed marked discrepancy between the whole cohort and elderly patients. Multivariate regression allowed for prediction of mortality with a sensitivity of 70% in the elderly as opposed to 46% in the whole cohort. Respective specificity was 90% vs. 95% (Fig. 4).

### Renal outcome

Renal outcomes are separated into CKD G5D and residual kidney function, based upon MDRD-eGFR.

#### CKD G5D and recovery

Overall, 35% (n = 29/82) of patients received KRT at initial admission (Fig. 2). There was a trend toward more frequent initial KRT in elderly (41% n = 22/54) as opposed to younger patients (25% *n* = 7/28; *p* = 0.157). Of those patients receiving KRT at diagnosis and surviving two years, 38% progressed to CKD G5D (*n* = 8/21). There was no difference in KRT recovery rate between elderly and younger patients (41% *n* = 9/22 and 57% *n* = 4/7; *p* = 0.451, respectively; Fig. 3e)). None of the patients that recovered from initial KRT-dependency progressed to CKD G5D in the observational period. None of the patients with sufficient renal function at diagnosis progressed to CKD G5D. However, display of KRT-frequencies and associated renal recovery rates in ten-year age steps, identified patients ≥ 80 years of age as a group with particularly dismal renal outcome (Fig. 3d). No patient in this group recovered from dialysis-dependency. All of these patients died within two years.

Univariate regression analysis revealed positive associations of initial eGFR and frequency of normal glomeruli and negative associations with KRT-dependency with CKD G5D-risk in the whole cohort (Tab. S4). However, in the elderly no histological parameter was associated with renal outcome. All patients that developed CKD G5D received KRT initially. Hence, no multivariate regression could be calculated because of quasi-separation of KRT as related to CKD G5D risk.

**Fig. 5:**
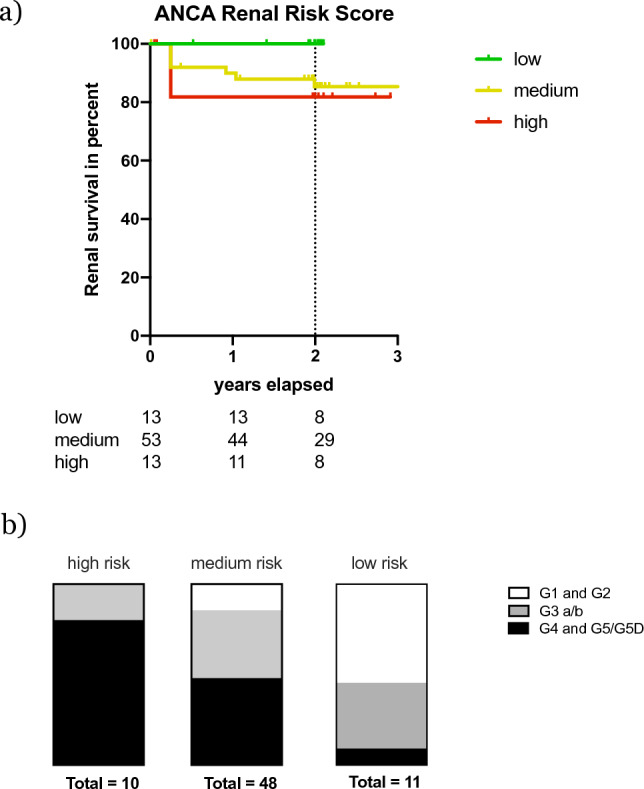
**a** Kaplan-Maier curve demonstrating renal survival of patients and number of patients at risk in their respective groups. Dashed lines indicate three and 24 months. Three months depict the earliest point in time to assign a patient to end stage renal disease. Grade of chronic kidney disease in the respective risk group (**b**)

Finally, we applied the ANCA-GN renal risk score (ARRS) [16] to our cohort (Fig. 5a). The low and medium risk group developed CKD G5D as predicted by the ARRS (renal survival proportions 100% and 85%, respectively). However, in the high-risk group (renal survival proportion 82%) we did not find a significant difference in CKD G5D risk as compared to the low or medium risk group in our cohort (high vs. low; *p* = 0.12). Analysis of KDIGO stages of chronic kidney disease, however, did show close association of the ARRS risk groups with severity of CKD stages (Fig. 5b).

#### Residual renal function

There was no difference in eGFR between elderly and younger patients who recovered from KRT, not in the first year (38.9 ml/min vs. 35.4 ml/min; *p* = 0.76 respectively), nor in the second (30.9 ml/min vs. 30.6 ml/min; *p* = 0.97, respectively; Fig. 3f). Patients who recovered from KRT showed significantly more necrotic glomeruli than patients who never received KRT (Tab. S5 and S6). These findings were similar in elderly and younger patients. Pearson’s correlation revealed multiple parameters associated with residual renal function after two years (Tab. S7), again with marked differences between elderly and younger patients and the whole cohort.

**Fig. 6:**
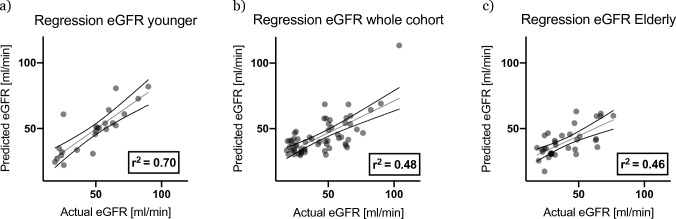
Multiple linear regression for eGFR after two years in the respective cohort. The coefficient of determination (*r*^2^) depicts a measure of association, with higher values indicating tighter association (in all data sets *p* < 0.0001). Independent variables: younger patients (serum albumin, age, initial eGFR, percentage of regular glomeruli) whole group (age, initial eGFR) and elderly patients (age, initial eGFR, haemoglobin level)

A multivariate linear regression model (Fig. 6) in younger patients yielded a reasonable coefficient of determination for eGFR after two years (*r*^2^ = 0.7). However, prognostic strength in the whole cohort and the elderly was low (*r*^2^ = 0.48 and *r*^2^ = 0.46, respectively).

## Discussion

This study supports previous findings that ANCA-associated vasculitis on the basis of a biopsy registry is a disease with high prevalence, not only in the whole cohort of indication renal biopsies, but especially in the elderly. It remains noteworthy however that frequencies of renal biopsy diagnoses display a derivative of (1) true incidence in the respective cohort, (2) hospital catchment area and (3) referral rate, (4) biopsy policy, and (5) disease group categorization.

In accordance with previously published data, our data show that AAV is the most common renal pathology finding, accounting for roughly 20% of all biopsies taken in a patient cohort ≥ 65 years of age [4, 17]. Additionally, we showed that AAV incidences rise constantly with age under the above mentioned caveats.

Our data show that AAV patients were typically diagnosed in a state of advanced renal damage. This finding could be attributable to the monosymptomatic (renal-limited) character of AAV [7, 18, 19] in the elderly, which was also discussed as a reason for lower initial BVAS scores and associated worse outcome of elderly patients.

In contrast to the MEPEX—protocol [20] the application of plasma exchange was accompanied, rather than superseded, by high-dose steroid therapy in our cohort. Importantly, we do not administer cumulative steroid doses of 3000 mg in the first three days to our patients, as we do not see a biological rationale as opposed to lower doses. It has been suggested that steroid reduction in patients aged 65 years or older suffices to control vasculitis [21]. In a recent nationwide prospective cohort study on steroid-dose reduction regimens in elderly subjects conducted in Japan, patients receiving lower doses experienced fewer infectious side effects without lower treatment efficacy [22]. Accordingly, in a recent study from the UK, steroid reduction in the elderly reduced infectious side effects without lowering therapy efficacy [23]. These findings might be attributable to lower AAV relapse rates in the elderly, as found in a recent study by the French vasculitis registry. Nevertheless, treatment with both steroids and antimetabolites further reduced relapse-rates also in the very elderly [24]. These data are in line with the EULAR/ERA-EDTA recommendations [25] and corroborate our findings of favourable outcomes following bivalent maintenance therapy in elderly patients.

Our data indicate that there is a substantial chance of recovery from KRT when patients are treated with the whole armamentarium of immunomodulatory treatments, including plasma exchange. In our study this also holds true for elderly patients. Of note, the status of plasma exchange has been profoundly questioned in a momentous investigation by Walsh and colleagues that did not show benefit for either renal or overall survival in patients with AAV [26]. Our data suggest that elderly as well as younger patients can effectively avoid progression to CKD G5D by guideline-directed therapy. In fact, also patients who received KRT at primary admission and recovered were able to maintain independent renal function. Although those patients persisted in an advanced stage of CKD, there was no difference between the elderly and younger patients with regard to eGFR. These data suggest that elderly patients benefit from therapy not only qualitatively (recovery rate) but also quantitatively (eGFR) to the same extent as younger patients.

For intensive treatment, prognostic models are crucial. Importantly, this study finds that renal and survival model accuracy differs dependent on age: while in the younger patient group the prediction of eGFR was reliable, the analysed clinic-pathological parameters failed to accurately predict renal outcome in the elderly. One possible explanation is that in elderly patients (as compared to younger patients) residual renal function is more likely a derivative of systemic conditions rather than renal limited determinants, e.g., heart failure as surrogate for cardio-renal syndrome or severe infectious complications leading to acute kidney injury not associated with AAV. In conclusion, our data on residual renal function indicate that routinely assessed parameters do not allow for reasonable prognosis in the elderly. Importantly, this implies that clinically intuitive “unfavourable predictors” should not inform decision making.

Application of the recently published ARRS to our cohort confirmed prognostic accuracy in the low and medium, yet not in the high-risk group. Possibly the two-year follow-up period did not suffice to elucidate the difference in renal survival. Indeed, the ARRS is validated with a follow-up of 36 months, and there were CKD G5D events in that latter phase in the original publication. Accordingly, we found a close association of the severity of CKD with the ARRS.

Most likely attributable to “fragility of results” we did not find a statistically significant difference in mortality between elderly and younger patients [27]. An age-group-wise analysis revealing that octogenarians are the group with distinct adverse mortality and renal outcomes was more informative. In the general population in Germany age above 80 years marks the end of the natural life span, nevertheless the life expectancy of people aged 80 years in Germany is roughly nine years [28]. On the one hand, it could be argued that on the basis of these data therapeutic alacrity should be tamed in order to impede unpleasant and eventually futile therapeutic attempts. On the other hand, these data imply that even in very elderly patients, there is a chance for almost one out of three patients to survive for at least two years. Upon deeper analysis, mortality in AAV appears as a two-peaked phenomenon. The first peak displays in the acute phase, in which patients die following severity of primary disease and concomitant factors, such as infectious complications or aggravating comorbidities. In the second mortality phase patients die putatively due to either relapsing AAV, therapy-associated complications or non-AAV-related reasons. In the elderly, one has to consider an altered ratio of disease vs. treatment burden and quality vs. length of life as opposed to younger patients. Therefore only “hard” medical endpoints might not suffice to inform indication for medical treatment. Thus, it would be of interest in further research to assess individual parameters with different treatment intensities in that particular patient subgroup with the highest burden of mortality.

Our study has limitations. Of utmost importance is the retrospective observational character of the study, which limits data on patients’ conditions and treatment decisions. As this study is a single centre investigation at a university hospital, our results might be difficult to generalize to other clinical situations. Our data are limited to patients with renal AAV, so we cannot draw conclusions for patients with extrarenal disease, milder forms and patients with contraindications to kidney biopsy. Finally, we lack data of adverse events, especially in terms of infectious complications of immunosuppression and relapse rates of patients as related to the outcomes of renal and overall survival.

## Conlcusions

To conclude, our data show, firstly, that under therapy there is no meaningful difference in outcome regarding renal function and survival in AAV patients up to 80 years of age as compared to younger patients in a two-year follow-up. Secondly, there might be no one-size-fits-all approach to renal or survival modelling. Hence, generally applied prognostication models might neglect vulnerable subgroups, as shown here in very elderly patients. Finally, research efforts beyond standard modelling and hard medical endpoints are needed to inform therapy indication and intensity in very elderly patients with AAV who carry the highest mortality and morbidity burden.

### Supplementary Information

Below is the link to the electronic supplementary material.

Supplementary file1 (PDF 67 KB)

Supplementary file2 (PDF 81 KB)

Supplementary file3 (PDF 56 KB)

Supplementary file4 (PDF 82 KB)

Supplementary file5 (PDF 67 KB)

Supplementary file6 (PDF 55 KB)

Supplementary file7 (PDF 58 KB)

Supplementary file7 (PDF 13 KB)

## Data Availability

All relevant data are reported in the article. Additional data can be provided by the corresponding author on request.

## References

[1] Harper L, Savage CO (2005). ANCA-associated renal vasculitis at the end of the twentieth century—a disease of older patients. Rheumatology.

[2] Watts RA, Lane SE, Bentham G, Scott DGI (2000). Epidemiology of systemic vasculitis a ten-year study in the United Kingdom. Arthritis Rheum.

[3] Pagnoux C, Hogan SL, Chin H (2008). Predictors of treatment resistance and relapse in antineutrophil cytoplasmic antibody-associated small-vessel vasculitis: comparison of two independent cohorts. Arthritis Rheum.

[4] Haris Á, Polner K, Arányi J, Braunitzer H, Kaszás I, Mucsi I (2014). Clinical outcomes of ANCA-associated vasculitis in elderly patients. Int Urol Nephrol.

[5] Weiner M, Goh SM, Mohammad AJ (2015). Outcome and treatment of elderly patients with ANCA-associated vasculitis. Clin J Am Soc Nephrol.

[6] Chen M, Yu F, Zhang Y, Zhao M (2008). Antineutrophil cytoplasmic autoantibody-associated vasculitis in older patients. Medicine (Baltimore).

[7] Bomback AS, Appel GB, Radhakrishnan J (2011). ANCA-associated glomerulonephritis in the very elderly. Kidney Int.

[8] Gavazzi G, Krause K-H (2002). Ageing and infection. Lancet Infect Dis.

[9] McGregor JAG, Negrete-Lopez R, Poulton CJ (2015). Adverse events and infectious burden, microbes and temporal outline from immunosuppressive therapy in antineutrophil cytoplasmic antibody-associated vasculitis with native renal function. Nephrol Dial Transplant.

[10] Yang L, Xie H, Liu Z (2018). Risk factors for infectious complications of ANCA-associated vasculitis: a cohort study. BMC Nephrol.

[11] Chung SA, Langford CA, Maz M (2021). American college of rheumatology/vasculitis foundation guideline for the management of antineutrophil cytoplasmic antibody-associated vasculitis. Arthritis Care Res (Hoboken).

[12] Brix SR, Noriega M, Tennstedt P (2018). Development and validation of a renal risk score in ANCA-associated glomerulonephritis. Kidney Int.

[13] de Groot K, Harper L, Jayne DRW (2009). Pulse versus daily oral cyclophosphamide for induction of remission in antineutrophil cytoplasmic antibody-associated vasculitis: a randomized trial. Ann Intern Med.

[14] Stone JH, Merkel PA, Spiera R (2010). Rituximab versus cyclophosphamide for ANCA-associated vasculitis. N Engl J Med.

[15] Guillevin L, Pagnoux C, Karras A (2014). Rituximab versus azathioprine for maintenance in ANCA-associated vasculitis. N Engl J Med.

[16] Brix SR, Noriega M, Tennstedt P (2018). Development and validation of a renal risk score in ANCA-associated glomerulonephritis. Kidney Int.

[17] Moutzouris D-A, Herlitz L, Appel GB (2009). Renal biopsy in the very elderly. Clin J Am Soc Nephrol.

[18] Jefferson JA (2015). Treating elderly patients with ANCA-associated vasculitis. Clin J Am Soc Nephrol.

[19] Hamour SM, Salama AD (2011). ANCA comes of age—but with caveats. Kidney Int.

[20] Jayne DRW, Gaskin G, Rasmussen N (2007). Randomized trial of plasma exchange or high-dosage methylprednisolone as adjunctive therapy for severe renal vasculitis. J Am Soc Nephrol.

[21] Pagnoux C, Quéméneur T, Ninet J (2015). Treatment of systemic necrotizing vasculitides in patients aged sixty-five years or older: results of a multicenter, open-label, randomized controlled trial of corticosteroid and cyclophosphamide-based induction therapy. Arthritis Rheumatol.

[22] Sada K-E, Ohashi K, Asano Y (2020). Treatment-related damage in elderly-onset ANCA-associated vasculitis: safety outcome analysis of two nationwide prospective cohort studies. Arthritis Res Ther.

[23] McGovern D, Williams SP, Parsons K (2020). Long-term outcomes in elderly patients with ANCA-associated vasculitis. Rheumatology.

[24] Thietart S, Beinse G, Smets P (2022). Patients of 75 years and over with ANCA-associated vasculitis have a lower relapse risk than younger patients: a multicentre cohort study. J Intern Med.

[25] Yates M, Watts RA, Bajema IM (2016). EULAR/ERA-EDTA recommendations for the management of ANCA-associated vasculitis. Ann Rheum Dis.

[26] Walsh M, Merkel PA, Peh C-A (2020). Plasma exchange and glucocorticoids in severe ANCA-associated vasculitis. N Engl J Med.

[27] Walsh M, Srinathan SK, McAuley DF (2014). The statistical significance of randomized controlled trial results is frequently fragile: a case for a fragility Index. J Clin Epidemiol.

[28] DeStatis. Durchschnittliche Lebenserwartung (Periodensterbetafel): Deutschland, Jahre, Geschlecht, Vollendetes Alter (18.11.2022/08:44:33). https://www-genesis.destatis.de/genesis/online?sequenz=tabelleErgebnis&selectionname=12621-0002&zeitscheiben=16&sachmerkmal=ALT577&sachschluessel=ALTVOLL000,ALTVOLL020,ALTVOLL040,ALTVOLL060,ALTVOLL065,ALTVOLL080#abreadcrumb. Accessed 18 Nov 2022

