# Supplementary material for: General prognostic models may neglect vulnerable subgroups in ANCA-associated vasculitis
Source: J Nephrol. 2023 Sep 28;36(8):2269–80. doi: 10.1007/s40620-023-01726-5 (PMC10638135; doi:10.1007/s40620-023-01726-5)
Supplement: Supplementary file 1 — Supplementary file1 (PDF 67 KB) [file 40620_2023_1726_MOESM1_ESM.pdf]

Table S1. Immunosuppressive therapy (without plasma exchange)

Induction therapy	Elderly	Younger	p
Cyclophosphamide + Steroids<br>(thereof Cyclophosphamide orally)	49<br>6	27<br>-	
Rituximab + Steroids	1	0	
**Σ (standard therapy)**	**50 (93%)**	**27 (96%)**	0.656
Steroids alone	2	0	
none	1	0	
unknown	1	1	
**Σ (alternative / none / unknown)**	**4 (7%)**	**1 (4%)**	
**Maintenance therapy**			
Azathioprine	14	4	
Mycophenolate	18	17	
Rituximab	1	1	
**Σ (standard therapy)**	**33 (67%)**	**22 (81%)**	0.284
Steroids alone	4	1	
Ciclosporine A	0	1	
Cyclophosphamide	1	0	
none	3	0	
unknown	(16%)	3 (11%)	
**Σ (alternative / none / unknown)**	**16 (33%)**	**5 (19%)**	

Depicted are absolute patient numbers (percentages in parenthesis) in the respective age cohort and category. Absolute patients numbers in the maintenance therapy are lower because of deceased patients that could not enter the maintenance therapy. P values refer to Fisher's exact test of standard vs. alternative therapy in the respective treatment period.
