# Supplementary material for: General prognostic models may neglect vulnerable subgroups in ANCA-associated vasculitis
Source: J Nephrol. 2023 Sep 28;36(8):2269–80. doi: 10.1007/s40620-023-01726-5 (PMC10638135; doi:10.1007/s40620-023-01726-5)
Supplement: Supplementary file 2 — Supplementary file2 (PDF 81 KB) [file 40620_2023_1726_MOESM2_ESM.pdf]

Table S2. Deceased patients

identifier	Age at diagnosis (years)	Comorbidity score	Time to event (days)	Female	KRT	CKD G5D*	DAH	ANCA target antigen	cause of death
T1	86	1	**4**	no	yes	n.a.	yes	MPO	infection
T2	86	2	234	yes	no	no	yes	MPO	TAVR complication
T3	85	1	393	no	no	no	no	MPO	lung fibrosis / AAV §
T4	84	4	**29**	no	yes	n.a.	yes	MPO	AAV
T5	82	2	659	yes	yes	yes	yes	PR3	not known
T6	82	3	**17**	yes	yes	n.a.	yes	MPO	AAV
T7	81	2	**3**	yes	yes	n.a.	no	PR3	AAV
T8	79	4	185	no	no	no	no	MPO	lung fibrosis / AAV §
T9	77	2	132	no	yes	yes	yes	PR3	infection
T10	72	2	412	yes	yes	yes	yes	MPO	infection after transplantation
T11	60	3	**4**	yes	yes	n.a.	yes	MPO	CABG / AAV
Mean or proportion [%]	79.5	2.3	189 (all) 336 (if survived ≥30 days)	55%	73%	50%°	73%	MPO 73%	AAV: 54% infection: 27%

Depicted are individual characteristics of deceased patients.
* CKD G5D could not be determined in patients that died less than 12 weeks after initial diagnosis (=n.a.)
° percentage of CKD G5D patients as fraction of patients at risk for CKD G5D, hence excluding "n.a." – patients.
§ lung fibrosis as a typical complication of AAV in the elderly, and hence was grouped into AAV-associated events.

Abbreviations: CABG, coronary artery bypass graft surgery; DAH, diffuse alveolar haemorrhage; CKD G5D, chronic kidney disease G5 treated by dialysis ; n.a., not applicable; KRT, kidney replacement therapy; TAVR, trans-catheter aortic valve replacement;
