# Supplementary material for: General prognostic models may neglect vulnerable subgroups in ANCA-associated vasculitis
Source: J Nephrol. 2023 Sep 28;36(8):2269–80. doi: 10.1007/s40620-023-01726-5 (PMC10638135; doi:10.1007/s40620-023-01726-5)
Supplement: Supplementary file 3 — Supplementary file3 (PDF 56 KB) [file 40620_2023_1726_MOESM3_ESM.pdf]

Table S3. two-year mortality (univariate regression)

		eGFR initial	KRT	age	comorbidity score	MPO positivity	female	c-reactive protein	Hb	Albumin
all patients	OR<br>CI	0.961<br>[0.906 – 1.019]	**6.222**<br>**[1.501 – 25.793]**	**1.247**<br>**[1.078 – 1.443]**	**2.548**<br>**[1.380 – 4.705]**	0.577<br>[0.049 – 6.734]	1.382<br>[0.368 – 4.946]	1.004<br>[0.998 – 1.010]	0.876<br>[0.607 – 1.265]	0.975<br>[0.881 – 1.072]
	p	0.185	**0.012**	**0.003**	**0.003**	0.661	0.619	0.239	0.481	0.604
Elderly	OR<br>CI	0.949<br>[0.873 – 1.032]	**4.511**<br>**[1.017 – 20.001]**	**1.486**<br>**[1.155 – 1.912]**	**2.091**<br>**[1.035 – 4.224]**	0.929<br>[0.200 – 4.306]	1.095<br>[0.277 – 4.325]	1.002<br>[0.996 – 1.009]	0.881<br>[0.597 – 1.299]	0.979<br>[0.885 – 1.082]
	p	0.219	**0.047**	**0.002**	**0.040**	0.925	0.897	0.461	0.522	0.675
Younger	OR<br>CI	-	-	-	-	-	-	-	-	-
	p	-	-	-	-	-	-	-	-	-

Depicted are odds ratios (OR) and 95% confidence intervals (CI) for the association of the respective parameter with death in a univariate logistic model. Due to scarcity of events (n=1), no calculations were carried out in the younger cohort.
