# Supplementary material for: General prognostic models may neglect vulnerable subgroups in ANCA-associated vasculitis
Source: J Nephrol. 2023 Sep 28;36(8):2269–80. doi: 10.1007/s40620-023-01726-5 (PMC10638135; doi:10.1007/s40620-023-01726-5)
Supplement: Supplementary file 4 — Supplementary file4 (PDF 82 KB) [file 40620_2023_1726_MOESM4_ESM.pdf]

Table S4. ESRD: univariate regression

		eGFR initial	KRT	age	MPO positivity	comorbidity score	female	c-reactive protein	Hb	Albumin	IF/TA	Glomeruli		
												normal	necrotic	scarred
all	OR<br>CI	**.501**<br>**[.315 - .796]**	∞	1.018<br>[.961 – 1.078]	.375<br>[.103 – 1.367]	.594<br>[.251 – 1.407]	.848<br>[0.210 – 3.434]	1.007<br>[1.000 – 1.014]	.722<br>[.478 – 1.091]	.902<br>[.803 . 1.014]	.996<br>[.947 – 1.048]	**.011**<br>**[.000 - .741]**	9.237<br>[.727 – 117.398]	1.648<br>[.083 – 32.870]
	p	**0.003**	-	0.553	0.137	0.236	0.818	0.063	0.122	0.085	.885	**0.036**	0.087	0.743
Elderly	OR<br>CI	**.426**<br>**[.266 – .802]**	∞	.936<br>[.804 – 1.090]	.241<br>[.051 – 1.154]	.406<br>[0.131 – 1.263]	.750<br>[.149 – 3.769]	1.005<br>[.997 – 1.012]	**.567**<br>**[.338 - .982]**	.918<br>[.808 – 1.043]	0.991<br>[.941 – 1.043]	0.027<br>[.000 – 2.347]	2.484<br>[.140 – 44.020]	4.801<br>[.178 – 129.22]
	p	**0.008**	-	0.395	0.075	0.119	0.727	0.248	**0.043**	0.188	0.725	0.113	0.535	0.350
Younger	OR<br>CI	-	-	-	-	-	-	-	-	-	-	-	-	-
	p	-	-	-	-	-	-	-	-	-	-	-	-	-

Depicted are odds ratios (OR) and 95% confidence intervals (CI) for the association of the respective parameter with CKD G5D in a univariate logistic model. Due to scarcity of events (n=2), no calculations were carried out in the younger cohort. ∞ = infinite / quasi-separational parameter.
