# Supplementary material for: General prognostic models may neglect vulnerable subgroups in ANCA-associated vasculitis
Source: J Nephrol. 2023 Sep 28;36(8):2269–80. doi: 10.1007/s40620-023-01726-5 (PMC10638135; doi:10.1007/s40620-023-01726-5)
Supplement: Supplementary file 5 — Supplementary file5 (PDF 67 KB) [file 40620_2023_1726_MOESM5_ESM.pdf]

Table S5. Elderly: KRT cohort with respective outcomes vs. non-KRT cohort

		deceased	p	CKD G5D	p	recovered	p	non-KRT
**age**	**[years]**	81 ± 5	**0.011**	73 ± 5	0.953	73 ± 5	0.648	75 ± 6
**comorbidity index**	**[dimensionless]**	2.3 ± 1.0	0.083	0.8 ± 0.8	**0.038**	2.2 ± 1.2	0.055	1.5 ± 0.9
**eGFR initial**	**[ml/min]**	10.3 ± 6.9	0.184	5.8 ± 1.6	**0.036**	9.0 ± 2.9	**0.001**	25.4 ± 22.4
**eGFR year 1**	**[ml/min]**	-	-	-	-	38.9 ± 18.1	0.743	38.2 ± 15.7
**eGFR year 2**	**[ml/min]**	-	-	-	-	30.9 ± 10.9	0.109	41.5 ± 17.1
**c-reactive protein**	**[mg/l]**	178 ± 106	0.721	160 ± 81	0.870	150 ± 138	0.165	100 ± 89
**IF/TA**	**[%]**	55 ± 18	0.191	36 ±15	0.636	43 ± 24	0.787	40 ± 24
**Glomeruli (necrotic)**	**[%]**	41 ± 34	0.991	35 ± 18	0.179	56 ± 33	**0.014**	32 ± 22
**Glomeruli (scarred)**	**[%]**	32 ± 32	0.767	41 ± 24	0.151	22 ± 23	0.587	27 ± 24
**Glomeruli (normal)**	**[%]**	27 ± 19	0.615	24 ± 18	0.822	21 ± 24	**0.028**	40 ± 22

Baseline characteristics and renal follow-up (eGFR 1st and 2nd year) in the elderly cohort, KRT- and non-KRT patients, respectively. p-values refer to evaluation of data right and left of their respective position. Data are presented as mean ± SD.

Table S6. Younger: KRT cohort with respective outcomes vs. non-KRT cohort

		deceased	p	CKD G5D	p	recovered	p	non-KRT
**age**	**[years]**	(60)	-	55 ± 1	0.644	53 ± 7	0.782	51 ± 14
**Comorbidity index**	**[dimensionless]**	(3)	-	0.5 ± 0.7	0.633	0.8 ± 0.5	0.943	0.7 ± 1.0
**eGFR initial**	**[ml/min]**	(32.4)	-	3.3 ± 1.1	0.399	11.2 ± 11.3	0.186	33.6 ± 31.9
**eGFR year 1**	**[ml/min]**	-	-	-	-	35.4 ± 16.3	0.121	57.0 ± 25.2
**eGFR year 2**	**[ml/min]**	-	-	-	-	30.6 ± 12.6	0.108	55.5 ± 21.9
**C-reactive protein**	**[mg/l]**	(333)	-	279 ± 72	0.227	162 ± 101	0.291	113 ± 79
**IF/TA**	**[%]**	(25)	-	-		-	-	34 ± 20
**Glomeruli (necrotic)**	**[%]**	(18)	-	95 ± 8	0.162	67 ± 21	**0.009**	37 ± 19
**Glomeruli (scarred)**	**[%]**	(27)	-	6 ± 8	0.233	24 ± 17	0.875	26 ± 24
**Glomeruli (normal)**	**[%]**	(55)	-	0	0.137	9 ± 6	**< 0.001**	37 ± 24

Baseline characteristics and renal follow-up (eGFR 1st and 2nd year) in the younger cohort, KRT- and non-KRT patients, respectively. p-values refer to evaluation of data right and left of their respective position. Because of scarcity of events in the deceased subgroup (n=1), no statistics were calculated. Because interstitial fibrosis and tubular atrophy could not be reported in two cases in the „CKD G5D“ and „recovered“ subgroup, no statistics were calculated. Data are presented as mean ± SD.
