# Supplementary material for: General prognostic models may neglect vulnerable subgroups in ANCA-associated vasculitis
Source: J Nephrol. 2023 Sep 28;36(8):2269–80. doi: 10.1007/s40620-023-01726-5 (PMC10638135; doi:10.1007/s40620-023-01726-5)
Supplement: Supplementary file 6 — Supplementary file6 (PDF 55 KB) [file 40620_2023_1726_MOESM6_ESM.pdf]

Table S7. eGFR after two years: correlation

		eGFR initial	KRT	age	MPO positivity	comorbidity score	female	c-reactive protein	Hb	Albumin	IF/TA	Glomeruli		
												normal	necrotic	scarred
**all**	Pearson's correlation	**.621**	**-.344**	**-.501**	**-.359**	**-.325**	-.033	.210	.181	.040	**-.528**	.247	-.004	-.247
	p	**< 0.001**	**0.007**	**< 0.001**	**0.004**	**0.011**	0.799	0.104	0.166	,772	**0.001**	0.059	0.893	0.067
**Elderly**	Pearson's correlation	**.563**	-.271	-.218	-.177	-.095	-.055	.218	**.453**	.103	**-.415**	.092	.048	-.146
	p	**< 0.001**	0.109	0.200	0.302	0.588	0.748	0.201	**0.006**	0.573	**0.044**	0.597	0.785	0.402
**Younger**	Pearson's correlation	**.653**	**-.398**	**-.645**	**-.452**	**-.422**	.040	.187	-.022	-.021	**-.782**	**.487**	-.146	-.370
	p	**< 0.001**	**0.044**	**< 0.001**	**0.021**	**0.032**	0.846	0.371	0.918	0.922	**0.004**	**0.007**	0.486	0.069
