# Supplementary material for: General prognostic models may neglect vulnerable subgroups in ANCA-associated vasculitis
Source: J Nephrol. 2023 Sep 28;36(8):2269–80. doi: 10.1007/s40620-023-01726-5 (PMC10638135; doi:10.1007/s40620-023-01726-5)
Supplement: Supplementary file 7 — Supplementary file7 (PDF 58 KB) [file 40620_2023_1726_MOESM7_ESM.pdf]

Figure S1.

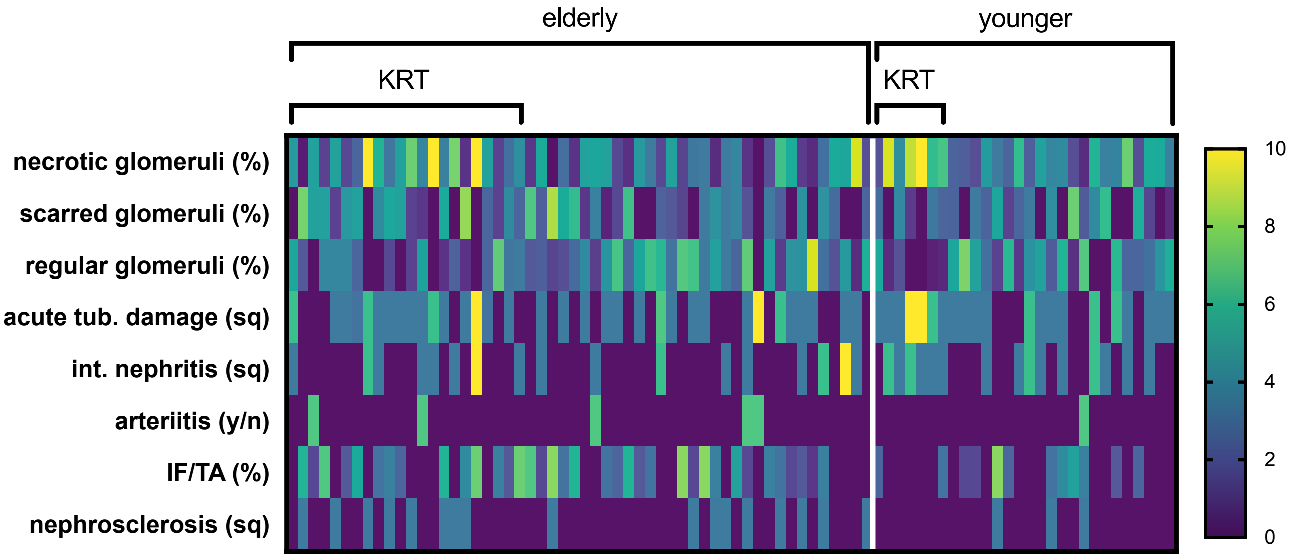

Depicted are histological criteria (rows) in respective individuals (columns). Patient data are shown in descending order of age from left to right, starting with patients treated by dialysis in the respective groups.

[sq] refers to "semiquantitative"; those dimensions have been rated in their extent from 0 (none) to 3 (severe). Those values have then been transformed in 0, 3,3; 6,6 and 10, respectively for easier comparability.
Arteriitis is stated as present (y) or not (n)

KRT, kidney replacement therapy; IF/TA interstitial fibrosis and tubular atrophy
