# Supplementary material for: General prognostic models may neglect vulnerable subgroups in ANCA-associated vasculitis
Source: J Nephrol. 2023 Sep 28;36(8):2269–80. doi: 10.1007/s40620-023-01726-5 (PMC10638135; doi:10.1007/s40620-023-01726-5)
Supplement: Supplementary file 8 — Supplementary file7 (PDF 13 KB) [file 40620_2023_1726_MOESM8_ESM.docx]

Lay summary

ANCA-associated vasculitis (AAV) is an organ and life-threatening disease that frequently affects elderly patients. The kidney is the most commonly affected organ. However, few studies have focussed on characteristics and treatment outcomes of elderly patients with biopsy-proven kidney involvement. In this retrospective observational study, we investigated the baseline and outcome characteristics of patients with AAV. Here, we asked the question if there were particular outcome differences in elderly and younger patients, dichotomized by the age of 65 years old. We found that elderly and younger patients have a comparable chance of renal recovery after guideline-directed treatment. Mortality was highest in patients above 80 years old. However, 30% of those patients survived in the two-year follow up after treatment.

We conclude that distinguishing patients by the age of 65, a common threshold to think of a person as being elderly, does not inform meaningful decision making. While we find that even in elderly patients there is a substantial chance of renal recovery and survival following treatment, we stress the fact that outcome predictors might substantially change between elderly and younger patients Additionally, we suggest further research to focus on patient-centred outcomes (e.g., quality of life, independence and autonomy) as compared to hard medical endpoints in the elderly to inform shared decision making of medical professionals and patients.
